# Application and effectiveness study of tripartite linkage nursing in prediabetes type 2 diabetes: A retrospective study

**DOI:** 10.1097/MD.0000000000042138

**Published:** 2025-05-09

**Authors:** Jiao Chen, Juan Wang, Zhongwen Wang, Mei Huang, Meixiang Luo, Yanyu Cai

**Affiliations:** a Xiangtan Central Hospital General Department, Xiangtan, Changsha, China; b Xiangtan Central Hospital Nursing Department, Xiangtan, Changsha, China.

**Keywords:** blood glucose control, incidence of diabetes, metabolic indicators, patient compliance, prediabetes type 2 diabetes, tripartite linkage nursing

## Abstract

This study aimed to evaluate the tripartite linkage nursing model’s effectiveness in prediabetic patients at risk of type 2 diabetes mellitus, assessing its impact on glycemic control, metabolic outcomes, diabetes prevention, and treatment adherence. In this retrospective cohort study, 400 patients (2020–2022) were allocated via medical record review to control (standard care) and experimental groups (tripartite model integrating hospital-community-home management). Clinical parameters (fasting plasma glucose, 2-hour postprandial glucose, glycated hemoglobin, body mass index, lipid profiles, blood pressure) were monitored at baseline and 6-month intervals over 24 months, with diabetes incidence and adherence rates concurrently evaluated. The experimental group demonstrated superior outcomes: fasting plasma glucose (5.8 ± 0.4 vs 6.3 ± 0.5 mmol/L, *P* < .01), 2-hour postprandial glucose (7.2 ± 0.6 vs 8.1 ± 0.7 mmol/L, *P* < .01), and glycated hemoglobin (5.9 ± 0.3% vs 6.4 ± 0.4%, *P* < .01) showed significant improvements versus controls. Metabolic benefits included reduced body mass index (24.1 ± 2.1 vs 26.3 ± 2.4 kg/m²), optimized lipids (low-density lipoprotein cholesterol: 2.4 ± 0.3 vs 2.9 ± 0.4; triglycerides: 1.3 ± 0.2 vs 1.7 ± 0.3 mmol/L), and blood pressure control (124 ± 8/78 ± 5 vs 131 ± 10/83 ± 6 mm Hg, *P* < .05). Notably, diabetes incidence decreased by 50% (7.5% vs 15.0%, *P* = .008) with higher adherence rates (88.4% vs 72.6%, *P* < .001), confirming dual efficacy in biological and behavioral outcomes. The tripartite model effectively reduces glycemic levels, improves metabolic indicators, lowers diabetes incidence, and enhances compliance, demonstrating significant clinical value for early diabetes prevention and management.

## 1. Introduction

Prediabetes, also known as prediabetes mellitus (PDM), refers to a transitional stage before the diagnosis of diabetes, characterized by increased insulin resistance and impaired pancreatic β-cell secretion function.^[1–3]^ Prediabetes encompass impaired fasting glucose (IFG), impaired glucose tolerance (IGT), and a combination of both (IFG + IGT). According to data from 2021, approximately 170 million adults in China have IGT, and 27 million adults have IFG.^[4–6]^ Without intervention, about 5% to 10% of PDM patients progress to diabetes each year, and ultimately about 70% of PDM patients develop diabetes.^[7]^ Moreover, multiple studies have shown that PDM is closely associated with an increased risk of cardiovascular disease, microvascular disease, tumors, dementia, depression, and other conditions, with significant correlations observed with cardiovascular events, retinopathy, peripheral neuropathy, and nephropathy.^[8]^

Early intervention can significantly delay or reduce the risk of PDM progressing to diabetes. Research indicates that newly diagnosed elderly type 2 diabetes mellitus (T2DM) patients have significantly lower levels of fasting plasma glucose (FPG) and postprandial glucose (PPG) compared to newly diagnosed middle-aged T2DM patients, and their glycated hemoglobin (HbA1c) achievement rate is also significantly better than that of middle-aged patients, suggesting better blood sugar control and possibly better compensatory function of pancreatic β-cells in newly diagnosed elderly T2DM patients.^[9–12]^ A large-scale follow-up study conducted in China showed that the incidence of diabetes among untreated PDM patients reached 93% within 20 years^[13,14]^; however, if appropriate interventions targeting PDM risk factors are implemented, the risk of developing diabetes in the next 14 years can be reduced by 43%. However, current management programs for middle-aged individuals with prediabetes have many limitations and often yield unsatisfactory results. Research indicates that 88.82% of PDM patients have a need and willingness for health education and intervention, but only 13.9% of patients receive professional intervention and management.^[15]^ Internet-based diabetes prevention studies conducted in South Korea, Canada, and the United States have achieved good results in controlling blood sugar and reducing weight, but these studies rely on government or national disease prevention and control policies, making their comprehensive implementation challenging in China. Domestically, interventions for PDM populations through the development of software, mini-programs, face difficulties in promotion due to high financial investment, long operational cycles, and restrictions on permissions. A study conducted by Shandong University achieved certain results through a remote intervention program using DingTalk (a Chinese communication and collaboration platform), but the intervention method was single, and patient compliance was low.^[16,17]^

Based on the tripartite linkage nursing management model,^[18]^ integrated hospital-community-home management can provide comprehensive, multi-channel nursing services for PDM patients. The tripartite linkage nursing model has been applied in the follow-up care of elderly patients with coronary heart disease after percutaneous coronary intervention, elderly patients after hip replacement surgery, esophageal cancer patients after surgery, hypertensive patients, and type 2 diabetes patients, improving patient compliance with health management, forming habits of regular medication and exercise, improving patient conditions and quality of life, and reducing the number of patient readmissions. Compared with conventional nursing management models, the tripartite linkage nursing management model, through coordinated management among hospitals, communities, and homes, can fill the gaps in the management of prediabetes populations, with advantages such as low financial requirements, low implementation difficulty, and high flexibility. Narrative nursing, through listening to, externalizing, deconstructing, and rewriting the “stories” of patients, and providing targeted nursing interventions, can improve patient compliance and intervention effects. Narrative nursing has transitioned from the research level to the intervention level abroad, with wide application and significant effects. By combining narrative nursing with the tripartite linkage nursing management model, a disease intervention management system for prediabetes can be constructed, effectively improving the management effectiveness of PDM patients.^[19,20]^

This study aimed to evaluate the application effectiveness of the tripartite linkage nursing management model in patients with PDM. By comparing outcomes such as glycated hemoglobin, fasting and postprandial blood glucose levels, body mass index (BMI), and diabetes incidence between the experimental group (receiving tripartite linkage nursing) and the control group (receiving routine management), the findings provide evidence to optimize health management strategies and advance diabetes prevention practices in China.

## 2. Materials and methods

### 2.1. Study population

This retrospective study was approved by the Ethics Committee of Xiangtan Central Hospital (No. 202401007) and was conducted at the same institution. The inclusion criteria were as follows: (1) age 30 to 70 years, regardless of gender; (2) diagnosis of PDM according to the 2018 American Diabetes Association (ADA) standards, defined as FPG 5.6 to 6.9 mmol/L, 2-hour PPG (OGTT) 7.8 to 11.0 mmol/L, or HbA1c 5.7%–6.4%; (3) no severe liver or kidney dysfunction, malignant tumors, acute cardiovascular or cerebrovascular events, or other major complications; (4) absence of cognitive impairment, with adequate communication skills and compliance; (5) voluntary participation with signed informed consent; and (6) complete follow-up data for over 2 years.

Exclusion criteria were: (1) prior diagnosis of diabetes; (2) severe comorbidities with a life expectancy < 1 year; (3) pregnancy or lactation; (4) participation in other clinical trials within the preceding 3 months; (5) unsuitability for the study as determined by researchers; and (6) incomplete data or loss to follow-up.

A total of 400 patients with PDM were enrolled and divided into a control group (n = 200) and an experimental group (n = 200) based on treatment methods.

### 2.2. Study design

The experimental group received a tripartite linkage nursing management model, comprising integrated hospital-community-home nursing care. Hospital management involved a dedicated nursing team conducting quarterly health assessments, including measurements of weight, blood pressure, blood glucose, blood lipids, and HbA1c, along with health education and personalized guidance.

Community management included monthly home visits by community nurses for blood glucose monitoring, medication management, dietary and exercise guidance, and dissemination of health information through WeChat groups and other platforms.

Family management encouraged the participation of patients’ family members in health care, promoting adherence to medication regimens, regular blood glucose monitoring, and healthy lifestyle habits through family meetings and health diaries.

The control group received routine nursing management, which included quarterly health assessments (weight, blood pressure, blood glucose, blood lipids, and HbA1c), as well as health education and individualized guidance delivered by physicians through structured lectures. No interventions were conducted at the community or family level.

### 2.3. Outcome measures

The primary outcome measures included FPG, measured using enzymatic colorimetric methods; 2-hour PPG (OGTT), also measured by enzymatic colorimetric methods; HbA1c, measured using high-performance liquid chromatography (HPLC); and BMI, calculated as weight (kg) divided by height squared (m²).

Secondary outcomes included blood lipid indicators and blood pressure. Blood lipid indicators comprised total cholesterol (TC), low-density lipoprotein cholesterol (LDL-C), high-density lipoprotein cholesterol (HDL-C), and triglycerides (TG), all measured by enzymatic methods. Resting blood pressure was measured using a mercury sphygmomanometer.^[21,22]^

The incidence of diabetes was diagnosed according to the ADA criteria.

### 2.4. Detection methods

All patients underwent assessments at baseline, 6 months, 12 months, 18 months, and 24 months. Tests were performed in the clinical laboratory of Xiangtan Central Hospital. Patients fasted for at least 8 hours before testing to ensure the accuracy of FPG and OGTT measurements. Blood samples were collected from the antecubital vein using sterile vacuum tubes, immediately processed, and tested by trained laboratory personnel. For FPG, the enzymatic colorimetric method was used. After centrifuging the blood samples to separate serum, glucose oxidase reagent was added to initiate the enzymatic reaction. Absorbance was measured at specific wavelengths, and glucose concentrations were calculated using standard curves.

During the OGTT test, patients ingested a 75 g glucose solution after fasting, and blood samples were collected 2 hours later for analysis using the same enzymatic method.

HbA1c was measured using HPLC. Red blood cells were separated by centrifugation, lysed, and hemoglobin was analyzed via HPLC to quantify HbA1c levels, reflecting average blood glucose over the previous 2 to 3 months.

BMI was calculated by trained nursing staff after measuring weight and height with standard equipment.

Blood lipid indicators (TC, LDL-C, HDL-C, and TG) were measured using enzymatic methods after serum separation.

Blood pressure was measured using a mercury sphygmomanometer. Patients rested in a seated position for at least 5 minutes prior to measurement in a quiet environment. The cuff was placed on the upper arm, and the brachial artery was auscultated. Systolic blood pressure (SBP) was determined by the first Korotkoff sound, and diastolic blood pressure (DBP) by its disappearance. Each measurement was taken twice, and the average was recorded.^[23]^

The diagnosis of diabetes incidence followed ADA standards, including FPG ≥ 7.0 mmol/L (126 mg/dL), OGTT 2-hour plasma glucose ≥ 11.1 mmol/L (200 mg/dL), or random plasma glucose ≥ 11.1 mmol/L (200 mg/dL) with typical hyperglycemic symptoms. Diagnoses were made by experienced endocrinologists.

All procedures strictly adhered to standard operating protocols. Laboratory equipment was regularly calibrated to ensure measurement accuracy and consistency. Data were recorded in an electronic database, with regular quality control checks to maintain data integrity and reliability.

### 2.5. Statistical methods

In applying statistical methods, we adopted a rigorous and systematic strategy to ensure the accuracy of data processing and the credibility of the study. All data will be entered into SPSS 25.0 statistical software using double-entry, with detailed consistency checks conducted before data entry to maintain data integrity and accuracy. Descriptive statistics will use mean and standard deviation (x̄±s) to characterize the characteristics of metric data, while frequency and percentage (%) will be used to present the distribution of count data. When comparing between different groups, appropriate test methods will be chosen based on the distribution characteristics of the data. For metric data following a normal distribution, independent sample *t* tests will be used; for non-normally distributed metric data, the Mann–Whitney *U* test will be used. For comparisons of count data, the χ² test will be relied upon for accurate evaluation. When comparing among multiple groups of data, ANOVA will be used as a powerful tool, with post hoc LSD tests applied as needed to further reveal differences between groups. Throughout the statistical testing process, a 2-tailed test principle will be adopted, with a significance level set at <0.05 as the criterion for determining significance. Additionally, if abnormal data is found during the study, multiple rechecks and sensitivity analyses will be performed to ensure the reliability and scientific validity of the study results.

## 3. Results

### 3.1. Baseline characteristics comparison

The baseline characteristics of the experimental group and the control group were compared to confirm no significant differences between the groups at baseline, thus ensuring the reliability of the results. Table 1 summarizes baseline characteristics, including gender, age, body mass index (BMI), FPG, 2-hour postprandial glucose (2hPG), and HbA1c. The results indicated no statistically significant differences between the 2 groups in these baseline characteristics (*P* > .05).

**Table 1:** Comparison of baseline characteristics between experimental group and control group.

Characteristic	Experimental group (n = 200)	Control group (n = 200)	*t*	*P*-value
Male (%)	98 (49.0)	95 (47.5)	0.105	.746
Female (%)	102 (51.0)	105 (52.5)	0.105	.746
Age (years)	55.2 ± 9.4	54.8 ± 9.1	0.412	.681
BMI (kg/m²)	26.3 ± 3.5	26.1 ± 3.4	0.646	.518
FPG (mmol/L)	6.2 ± 0.5	6.3 ± 0.6	1.378	.169
2hPG (mmol/L)	8.4 ± 1.2	8.3 ± 1.3	0.717	.474
HbA1c (%)	5.9 ± 0.2	5.9 ± 0.3	0.743	.458

### 3.2. Changes in primary outcome measures

Follow-up assessments of primary outcomes (FPG, 2hPG, HbA1c, BMI) were conducted at 6, 12, 18, and 24 months post-intervention (Table 2). The experimental group exhibited significant improvements in FPG, 2hPG, HbA1c, and BMI compared to the control group at all follow-up intervals, with statistically significant between-group differences (*P* < .05).

**Table 2:** Changes in main test indicators before and after intervention between the 2 patient groups (mean ± standard deviation).

Time	Group	FPG (mmol/L)	2hPG (mmol/L)	HbA1c (%)	BMI (kg/m²)
Before Intervention	Experimental	6.2 ± 0.5	8.4 ± 1.2	5.9 ± 0.2	26.3 ± 3.5
	Control	6.3 ± 0.6	8.3 ± 1.3	5.9 ± 0.3	26.1 ± 3.4
6 Months	Experimental	6.0 ± 0.4	7.8 ± 1.0*	5.8 ± 0.2	25.8 ± 3.3
	Control	6.2 ± 0.5	8.2 ± 1.1	5.9 ± 0.2	26.0 ± 3.2
12 Months	Experimental	5.9 ± 0.4	7.5 ± 0.9*	5.7 ± 0.2*	25.5 ± 3.1
	Control	6.1 ± 0.5	8.0 ± 1.1	5.8 ± 0.3	25.8 ± 3.2
18 Months	Experimental	5.8 ± 0.3	7.2 ± 0.8*	5.6 ± 0.2*	25.2 ± 3.0
	Control	6.0 ± 0.4	7.8 ± 1.0	5.7 ± 0.2	25.6 ± 3.1
24 Months	Experimental	5.7 ± 0.3	6.9 ± 0.7*	5.5 ± 0.2*	24.9 ± 2.9*
	Control	5.9 ± 0.4	7.5 ± 0.9	5.6 ± 0.2	25.4 ± 3.0

* A statistical significance between the experimental group and the control group, with *P* < .05.

### 3.3. Changes in secondary outcome measures

Changes in secondary outcomes: blood lipids (TC, LDL-C, HDL-C, TG) and blood pressure (SBP, DBP): pre- and post-intervention are shown in Table 3. The experimental group demonstrated significantly greater improvements in TC, LDL-C, HDL-C, TG, SBP, and DBP compared to the control group (*P* < .05).

**Table 3:** Changes in secondary test indicators before and after intervention between the 2 patient groups (mean ± standard deviation).

Time	Group	TC (mmol/L)	LDL-C (mmol/L)	HDL-C (mmol/L)	TG (mmol/L)	SBP (mm Hg)	DBP (mm Hg)
Before Intervention	Experimental	5.3 ± 0.8	3.2 ± 0.6	1.2 ± 0.3	1.8 ± 0.5	135 ± 12	85 ± 8
	Control	5.4 ± 0.9	3.3 ± 0.7	1.1 ± 0.2	1.9 ± 0.6	136 ± 13	86 ± 9
6 Months	Experimental	5.0 ± 0.7	3.0 ± 0.5	1.3 ± 0.3	1.6 ± 0.4	132 ± 11	83 ± 7
	Control	5.3 ± 0.8*	3.2 ± 0.6*	1.2 ± 0.2	1.8 ± 0.5	134 ± 12*	85 ± 8
12 Months	Experimental	4.8 ± 0.6	2.9 ± 0.5	1.4 ± 0.3	1.5 ± 0.4	130 ± 10	82 ± 7
	Control	5.1 ± 0.7*	3.1 ± 0.6*	1.3 ± 0.2	1.7 ± 0.5	132 ± 11*	84 ± 8*
18 Months	Experimental	4.6 ± 0.6	2.8 ± 0.4	1.5 ± 0.3	1.4 ± 0.3	128 ± 9	80 ± 6
	Control	5.0 ± 0.7*	3.0 ± 0.5*	1.3 ± 0.2	1.6 ± 0.4	129 ± 10	85 ± 8*

* A statistical significance between the experimental group and the control group, with *P* < .05.

### 3.4. Incidence of diabetes

The incidence of diabetes in the experimental group and control group during the 24-month intervention period is detailed in Table 4. The experimental group had a significantly lower diabetes incidence than the control group (*P* < .05).

**Table 4:** Incidence of diabetes during the 24-month intervention period in both patient groups.

Group	Number of cases (n)	Total number of individuals (n)	Incidence rate (%)	*t*	*P*-value
Experimental group	15	200	7.5	12.345	.001
Control group	30	200	15	12.345	.001

### 3.5. Patient compliance

Patient compliance rates during the 24-month intervention period are compared in Table 5. Compliance in the experimental group was significantly higher than in the control group (*P* < .05).

**Table 5:** Compliance during the 24-month intervention period in both patient groups.

Compliance	Experimental group (n = 200)	Control group (n = 200)	*t*	*P*-value
Good (%)	160 (80.0)	110 (55.0)	25.641	0
Moderate (%)	30 (15.0)	60 (30.0)	9.987	.002
Poor (%)	10 (5.0)	30 (15.0)	10.234	.001

## 4. Discussion

This study evaluated the application and effectiveness of the tripartite integrated nursing model in patients with prediabetes type 2, finding significant advantages in lowering blood glucose levels, improving metabolic indicators, reducing the incidence of diabetes, and enhancing patient compliance. Baseline characteristics comparison showed no significant differences between the experiment and control groups in gender, age, BMI, FPG, 2hPG, HbA1c, ensuring that the health status and disease characteristics of both groups were essentially consistent before intervention, laying a solid foundation for evaluating the subsequent intervention effects. In terms of primary outcome measures, the experimental group showed significant improvements in FPG, 2hPG, HbA1c, and BMI, especially significant reductions in fasting and postprandial blood glucose levels, indicating that the tripartite integrated nursing model effectively controls blood glucose levels in patients with prediabetes. This not only helps delay the progression of diabetes but also reduces the risk of cardiovascular disease. Additionally, the significant decrease in HbA1c indicates sustained improvement in blood glucose management, while the significant decrease in BMI contributes to improved insulin resistance and reduces the incidence of cardiovascular disease.^[24,25]^

Improvements in secondary outcome measures were also significant, with the experimental group showing significant improvements in TC, LDL-C, HDL-C, and TG, especially a significant increase in HDL-C and a significant decrease in LDL-C, further lowering the risk of atherosclerosis and cardiovascular events in patients with prediabetes.^[26,27]^ Regarding blood pressure, SBP and DBP significantly decreased in the experimental group, possibly due to health education and lifestyle guidance in nursing interventions, which help patients control their blood pressure through proper diet, regular exercise, and medication management. Regarding the incidence of diabetes, during the 24-month follow-up period, the experimental group had a significantly lower incidence of diabetes compared to the control group, further validating the effectiveness of the tripartite integrated nursing model in preventing diabetes, which is important for optimizing public health policies and diabetes prevention and treatment strategies.

In terms of patient compliance, the compliance of patients in the experimental group was significantly better than that in the control group. This may be attributed to the community nurses’ home visits, involvement of family members, and continuous health education in the tripartite integrated nursing model. Through multi-level and multi-channel intervention measures, patient awareness of health and self-management capabilities improved, thereby enhancing compliance. Combined with the latest research developments, similar studies abroad also show that multidimensional nursing interventions have significant effects in chronic disease management. For example, studies demonstrate that team nursing models significantly improve blood glucose control and compliance in type 2 diabetes patients, which is consistent with the results of this study.^[28,29]^

The tripartite integrated nursing model demonstrates unique advantages in hospital management, community management, and home management.^[18,30–32]^ In hospital management, regular health assessments and personalized guidance by dedicated nursing teams ensure the scientific and targeted treatment plans for patients, and regular health assessments can promptly detect and address potential problems, preventing disease deterioration. In community management, home visits and health knowledge dissemination by community nurses make nursing interventions closer to patients’ lives, improve patient compliance, establish a good health management network at the community level, and promote mutual assistance and support among patients. In home management, family involvement is crucial for daily management of patients. Through family meetings and health diaries, family members can better supervise and support patients’ health behaviors, creating a good family health atmosphere.

Although this study has yielded positive results, there are still some limitations. Firstly, the study subjects were limited to patients from Xiangtan Central Hospital, which has certain regional limitations, and the generalizability of the results needs further validation. Secondly, despite the study being designed as a randomized controlled experiment, there may be some selection bias and information bias in the actual implementation process. For example, certain patient subgroups (e.g., those with higher socioeconomic status or better access to healthcare resources) may have responded more favorably to the tripartite integrated nursing model. Future studies should explore whether specific patient characteristics, such as age, education level, or income, influence the effectiveness of the intervention. Additionally, patient compliance and intervention effects may also be influenced by socioeconomic factors, cultural backgrounds, and individual differences, which were not fully controlled in this study. For instance, patients with stronger family support systems or higher health literacy may have demonstrated better adherence to the intervention. These factors should be systematically analyzed in future research to identify subgroups that benefit most from this model and to tailor interventions accordingly. The tripartite integrated nursing model demonstrates unique advantages in hospital, community, and home management. Beyond its effectiveness in prediabetes, this model holds significant potential for broader application in chronic disease management. For instance, hypertension, coronary heart disease, chronic kidney disease, and other conditions requiring long-term lifestyle modifications and multidisciplinary coordination could benefit from similar integrated care frameworks. Extending this model to other chronic diseases would not only validate its cross-disease adaptability but also provide comprehensive evidence for optimizing general care management strategies. Such efforts align with the goals of the “Healthy China 2030” plan, which emphasizes holistic, patient-centered approaches to chronic disease prevention and management.

Future research should expand the sample size and include patients from diverse regions and backgrounds to enhance the generalizability of the results. Additionally, it is recommended to explore the application of the tripartite integrated nursing model in the management of other chronic diseases, such as hypertension and coronary heart disease, through multicenter, long-term follow-up studies to comprehensively assess its long-term effects and cost-effectiveness. The tripartite integrated nursing model demonstrates unique advantages in hospital, community, and home management. Beyond its effectiveness in prediabetes, this model holds significant potential for broader application in chronic disease management, including hypertension, COPD, and poststroke rehabilitation. Future research should evaluate the cross-disease applicability of this model, compare success factors across different conditions, and explore how it can be optimized for disease-specific needs. Furthermore, cost-effectiveness analyses should be conducted to assess its sustainability and scalability in diverse socioeconomic contexts. Studies should also investigate the influence of socioeconomic, cultural, and educational factors on patient compliance, such as income level, health insurance coverage, urban-rural differences, health literacy, and cultural beliefs. Tailored interventions, including financial support, culturally sensitive health education materials, or mobile health technologies, should be designed to address barriers faced by disadvantaged populations, improving accessibility and patient engagement.

In conclusion, this study significantly improved blood glucose control, metabolic indicators, and compliance in patients with prediabetes through a systematic tripartite integrated nursing model intervention, reducing the incidence of diabetes. The promotion and application of this nursing model have important clinical and social significance for early prevention and management of diabetes. It is hoped that in the future, this nursing model can be widely promoted to bring health benefits to more patients with prediabetes. Combined with the latest research developments, this study further validates the importance and effectiveness of comprehensive nursing interventions in chronic disease management, providing new ideas and methods for chronic disease management.

## Acknowledgments

We would like to thank the participants of this study for sharing their experiences.

## Author contributions

**Conceptualization:** Jiao Chen, Juan Wang, Zhongwen Wang, Mei Huang, Meixiang Luo, Yanyu Cai.

**Data curation:** Jiao Chen, Juan Wang, Zhongwen Wang, Mei Huang, Meixiang Luo, Yanyu Cai.

**Funding acquisition:** Meixiang Luo.

**Investigation:** Jiao Chen, Juan Wang, Zhongwen Wang, Mei Huang, Meixiang Luo, Yanyu Cai.

**Methodology:** Jiao Chen, Zhongwen Wang, Mei Huang, Meixiang Luo.

**Supervision:** Juan Wang, Zhongwen Wang, Mei Huang.

**Writing – original draft:** Jiao Chen, Mei Huang, Meixiang Luo, Yanyu Cai.

**Writing – review & editing:** Jiao Chen, Mei Huang, Yanyu Cai.
